# Progressive Skeletal Muscle Loss After Surgery and Adjuvant Radiotherapy Impact Survival Outcomes in Patients With Early Stage Cervical Cancer

**DOI:** 10.3389/fnut.2021.773506

**Published:** 2022-01-20

**Authors:** Jie Lee, Jhen-Bin Lin, Tze-Chien Chen, Ya-Ting Jan, Fang-Ju Sun, Yu-Jen Chen, Meng-Hao Wu

**Affiliations:** ^1^Department of Radiation Oncology, MacKay Memorial Hospital, Taipei, Taiwan; ^2^Department of Medicine, MacKay Medical College, New Taipei City, Taiwan; ^3^Department of Radiation Oncology, Changhua Christian Hospital, Changhua, Taiwan; ^4^Department of Obstetrics and Gynecology, MacKay Memorial Hospital, Taipei, Taiwan; ^5^Department of Radiology, MacKay Memorial Hospital, Taipei, Taiwan; ^6^Department of Biomedical Imaging and Radiological Sciences, National Yang Ming Chiao Tung University, Taipei, Taiwan; ^7^Department of Medical Research, MacKay Memorial Hospital, Taipei, Taiwan; ^8^Institute of Biomedical Informatics, National Yang Ming Chiao Tung University, Taipei, Taiwan

**Keywords:** skeletal muscle loss, pelvic radiotherapy, cervical cancer, nutrition, clinical outcome

## Abstract

The effect of skeletal muscle loss associated with surgery and adjuvant radiotherapy on survival outcomes in patients with early-stage cervical cancer remains unclear. We analyzed the data of 133 patients with early-stage cervical cancer who underwent surgery and adjuvant radiotherapy between 2013 and 2018 at two tertiary centers. Skeletal muscle changes were measured using computed tomography scans at baseline, at simulation for radiotherapy, and at 3 months post-treatment. A decrease of ≥5% in the skeletal muscle was defined as “muscle loss.” The Patient-Reported Outcome version of the Common Terminology Criteria for Adverse Events (PRO-CTCAE) was used to assess gastrointestinal toxicity. The Patient-Generated Subjective Global Assessment (PG-SGA) was used for nutritional assessment. Predictors of overall survival were identified using the Cox regression models. The median follow-up period was 3.7 years. After treatment, 32 patients (24.1%) experienced muscle loss. The rate of muscle loss was higher in patients with PRO-CTCAE score ≥3 or PG-SGA score ≥4 at the end of radiotherapy than in patients with PRO-CTCAE score ≤2 or PG-SGA score 0–3 (75.0 vs. 10.5%, *p* < 0.001; 71.4 vs. 2.2%, *p* < 0.001). The 3-year overall survival was significantly lower in patients with muscle loss than in those with muscle preserved (65.6 vs. 93.9%, *p* < 0.001). Multivariate analysis showed that muscle loss was independently associated with poor overall survival (hazard ratio, 4.55; 95% confidence interval: 1.63–12.72; *p* < 0.001). Muscle loss after surgery and adjuvant radiotherapy was associated with poor overall survival in patients with early-stage cervical cancer. Muscle loss is associated with patient-reported gastrointestinal toxicity and deterioration in nutritional status.

## Introduction

Cervical cancer is the fourth most commonly occurring cancer and the fourth leading cause of cancer-related deaths in women, with an estimated 604,000 new cases and 342,000 deaths worldwide in 2020 (1). Radical hysterectomy with bilateral pelvic lymph node dissection is the primary treatment for patients with International Federation of Gynecology and Obstetrics (FIGO) stage IB-IIA cervical cancer. Despite favorable outcomes after surgery, patients with risk factors for recurrence are administered adjuvant pelvic radiotherapy to reduce the risk of pelvic recurrence, although no significant improvement in overall survival due to adjuvant pelvic radiotherapy has been reported (2–4). Consideration of treatment-related morbidity is important.

Pelvic radiotherapy is associated with gastrointestinal (GI) toxicities that can be challenging for the patients, interfere with the quality of life, and lead to deterioration of nutritional status (5–14). Patients who experience a high symptom burden and deterioration of nutritional status might develop adverse changes in body composition, such as skeletal muscle loss (15–17). The skeletal muscle acts as an endocrine organ that produces and releases myokines, which play a role in regulating the metabolism and inflammation in the entire body (18). Studies have reported that skeletal muscle loss during chemoradiotherapy is associated with poor survival outcomes in patients with locally advanced cervical cancer (7–13). However, the effect of skeletal muscle loss associated with surgery and adjuvant radiotherapy on survival outcomes in patients with early-stage cervical cancer remains unclear.

**Figure 1:**
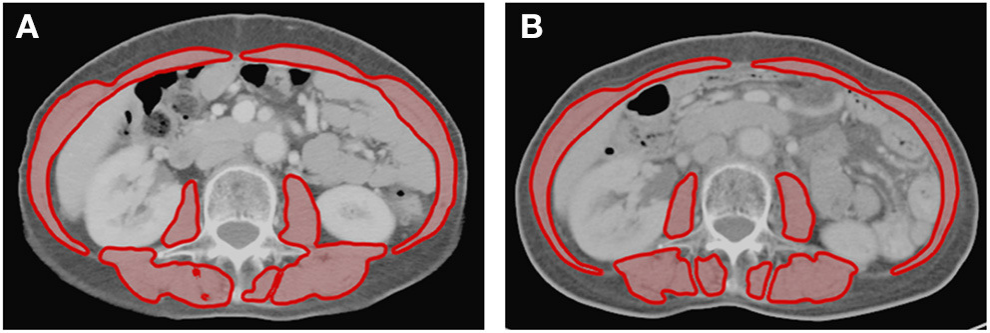
Axial cross-sectional areas of skeletal muscle (red) on CT images at the L3 vertebral level **(A)** before and **(B)** after treatment in one patient. The skeletal muscle areas of this patient were 81.6 and 77.3 cm^2^ before and after treatment, respectively. This patient had a reduction of 5.3% of skeletal muscle after treatment.

Skeletal muscle mass can be evaluated by a variety of techniques and reported as total body skeletal muscle mass, as appendicular skeletal muscle mass, or as muscle cross-sectional area of specific muscle groups or body locations (19). Computed tomography (CT) images are widely performed in cancer patients for routine cancer care and can provide objective skeletal muscle measurement. The cross-sectional areas of the skeletal muscle at the level of the third lumbar vertebra (L3) are strongly correlated with the total body skeletal muscle (20–22). The prognostic value of CT-based body composition measurement had also been evaluated and validated in various malignancies (23). Longitudinal analysis of CT images of cancer patients may help evaluate skeletal muscle changes during cancer treatments and their associations with clinical outcomes (Figure 1).

We hypothesized that skeletal muscle loss after surgery and adjuvant radiotherapy would affect survival outcomes in patients with early-stage cervical cancer. This study aimed to evaluate skeletal muscle using CT scans performed during routine cancer care and determine whether skeletal muscle loss is associated with survival outcomes in patients with early-stage cervical cancer.

## Patients, Materials, and Methods

### Patients

This study was approved by the Institutional Review Board. The need for informed consent was waived because of the retrospective and observational nature of the study. The data of patients with FIGO stage IB-IIA cervical cancer with indications for postoperative radiotherapy after hysterectomy between 2013 and 2018 were reviewed at two tertiary centers. The inclusion criteria were as follows: (a) adequate clinical data, GI toxicity data, and nutritional assessment data, (b) CT scans performed before surgery and within 3 months after adjuvant radiotherapy. Patients were excluded from the analysis if they had a history of other malignancies.

### Treatments

Pre-treatment CT scans were routinely performed for the pre-surgical workup. The surgeries were performed by accredited gynecologic oncologists, and included hysterectomy, bilateral salpingo-oophorectomy, and pelvic lymphadenectomy. After surgery, the patients were recommended adjuvant pelvic radiotherapy considering the pathological risk factors (tumor size, depth of cervical stromal invasion, and invasion of the lymphovascular space). For patients with pelvic lymph node metastasis, parametrial involvement, or positive surgical margins, adjuvant pelvic radiotherapy concurrent with cisplatin-based chemotherapy was indicated. After surgical wound healing, a CT scan was performed for planning radiotherapy. Pelvic radiotherapy was administered using intensity-modulated radiotherapy (IMRT) up to 45–50.4 Gy. The clinical target volume encompassed the obturator, internal iliac, external iliac, common iliac, and presacral nodal regions, and the upper vagina. Vaginal cuff brachytherapy was considered at the discretion of the treating physicians after the completion of pelvic IMRT. High-dose rate brachytherapy at 5 Gy for 4 fractions was delivered. Post-treatment CT scans were performed within 3 months after completion of radiotherapy.

### GI Toxicity Assessment

GI toxicities were assessed weekly using the Patient-Reported Outcome version of the Common Terminology Criteria for Adverse Events (PRO-CTCAE). The PRO-CTCAE questionnaires included the severity of abdominal pain, interference of abdominal pain with daily activities, and frequency of diarrhea, were administered to patients (24). Patients scored these three PRO-CTCAE items at home or recorded whenever severe or bothersome symptoms occurred. The PRO-CTCAE questionnaires were provided by patients to nurses in the health education room before weekly clinic appointments. The PRO-CTCAE scores toxicity on a 5-point Likert scale, with 0 indicating none, not at all, and never, respectively. We analyzed the highest score for each item during 3–5 weeks of radiotherapy because radiotherapy-induced GI toxicities generally become symptomatic at 3 weeks and reach a maximum at 5 weeks (25).

Physicians also graded the GI toxicity every week using CTCAE version 4.0. Previous studies reported that PRO-CTCAE could evaluate the treatment-related toxicity more accurately than the physician-graded CTCAE (6, 15). In this study, PRO-CTCAE data were used for analysis.

### Nutritional Assessment

We evaluated the nutritional status of patients using Patient-Generated Subjective Global Assessment (PG-SGA) at the beginning and end of radiotherapy. The PG-SGA provides a score (higher score indicates a higher risk of malnutrition) and categorizes patients into three distinct classes of nutritional status: A, well-nourished; B, suspected malnutrition or moderately malnourished; and C, severely malnourished. In this analysis, patients were categorized into two groups: well-nourished (PG-SGA score 0–3) and malnourished (PG-SGA score ≥4) (15, 26–28).

### Skeletal Muscle Measurement

**Figure 2:**
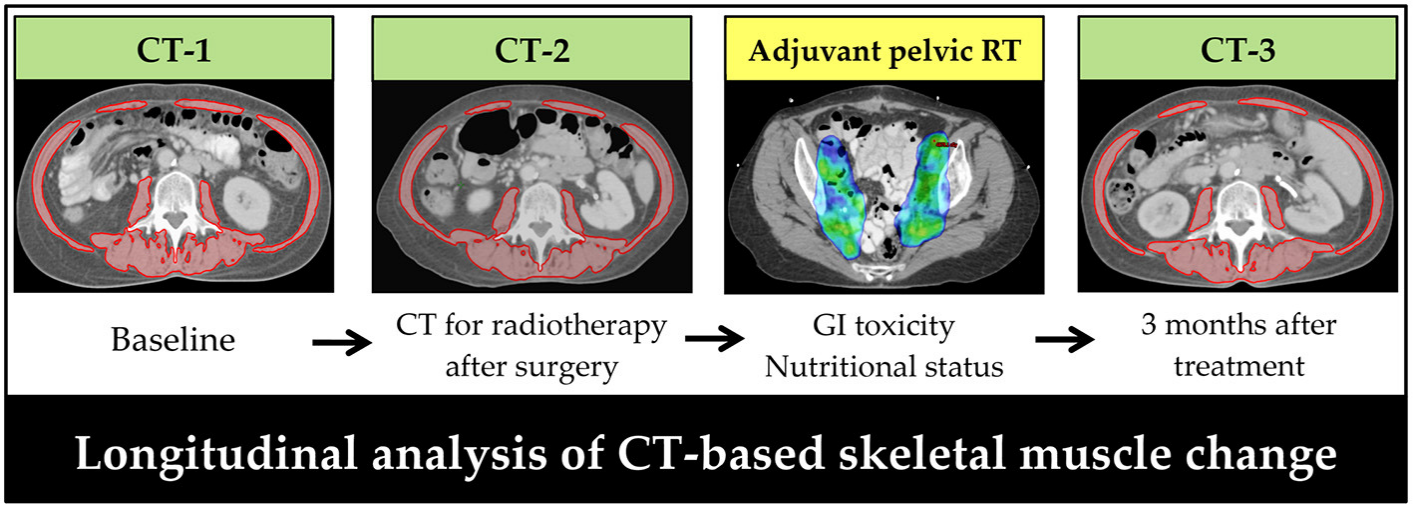
Timeline of computed tomography scans for patients with early-stage cervical cancer receiving surgery and post-operative pelvic radiotherapy. Skeletal muscle was assessed on a transversal computed tomography slice at the level of L3. Red: skeletal muscle area. CT, computed tomography; RT, radiotherapy.

The CT scans at three timepoints were retrieved for analysis (Figure 2). The cross-sectional area (cm^2^) of the skeletal muscle was measured on a single slice of the CT scan at the third lumbar vertebral level. One researcher, blinded to the patient information, measured the skeletal muscle using the Varian Eclipse software (Varian Medical Systems Inc., Palo Alto, CA, USA) (20–22, 29–31). Skeletal muscle was defined based on Hounsfield unit (HU) thresholds ranging from −29 to +150 HU. The skeletal muscle index (SMI) was calculated as the cross-sectional muscle area divided by height in square meters (cm^2^/m^2^) (32). The cut-off values for sarcopenia were set at the lowest tertile for SMI based on previous studies (33–36). The body mass index (BMI) within 2 weeks of the CT scans was obtained from medical records.

The current definition of cachexia is weight loss >5% over the past 6 months (17). Based on this cut-off value, several studies have reported that weight or muscle loss >5% during cancer treatment is associated with poor survival outcomes in cancer patients (35–38). In this study, patients with a decrease in BMI or SMI ≥5% after surgery and adjuvant radiotherapy were categorized as having weight loss or muscle loss, and those with a gain or decrease of <5% in BMI or SMI were categorized as “preserved”.

### Statistical Analysis

Continuous variables are expressed as medians and interquartile range (IQR) or mean ± standard deviation. The comparisons of continuous variables were analyzed using independent *t*-tests or Mann–Whitney *U* tests, as appropriate. Categorical data are expressed as frequency (%) and were analyzed using the chi-square test or Fisher's exact test. Changes in BMI and SMI were analyzed by repeated-measures ANOVA with Bonferroni adjustment for the *post-hoc* tests. Paired *t*-tests were used to assess changes in PG-SGA score between the start and the end of radiotherapy. McNemar's test was used to test for significant differences in the paired categorical data. Spearman's correlation coefficient was used to evaluate the correlations.

Overall survival (OS) and disease-free survival (DFS) were measured from the date of surgery to the date of death/last follow-up and the date of disease recurrence, death, or last follow-up, respectively. Univariate and multivariate analyses of OS and DFS were performed using the Cox proportional hazards model, and the results are presented as hazard ratios (HRs) with 95% confidence intervals (CIs). Multivariate models were selected by backward elimination with a significance level of 0.05. The data were analyzed using IBM SPSS software (version 21.0; IBM Corp., Armonk, NY, USA). Statistical significance was set at *p* < 0.05.

## Results

### Patient Characteristics

We reviewed the data of 181 patients with cervical cancer who underwent hysterectomy and adjuvant pelvic radiotherapy. Patients with a history of other malignancy (*n* = 4), missing PG-SGA data (*n* = 11), missing PRO-CTCAE data (*n* = 25), and in whom CT was not performed after treatment (*n* = 8) were excluded. The final analysis was included the data of 133 patients. The patient and tumor characteristics are summarized in Table 1. The median follow-up period was 3.7 years (IQR: 2.6–5.7), during which 22 (16.5%) patients experienced recurrence, and 17 (12.8%) patients died.

**Table 1:** Patient and tumor characteristics.

**Characteristics**	**Overall (*n* = 133)**
Age (years)	53 (46–61)
Stage (FIGO 2018)	
IB1	16 (12.0)
IB2	58 (43.6)
IB3	23 (17.3)
IIA1	26 (19.5)
IIA2	10 (7.5)
Histology	
Squamous cell carcinoma	104 (78.2)
Adenocarcinoma	29 (21.8)
Pathological cervical tumor size	
<4 cm	92 (69.2)
≥4 cm	41 (30.8)
Pathological risk factors	
Pelvc lymph node metastasis	41 (30.8)
Parametrial invasion	16 (12.0)
Positive surgical margin	8 (6.0)
Lymphovascular space invasion	103 (77.4)
Deep one-third cervical stromal invasion	97 (72.9)
Adjuvant treatment	
Radiotherapy only	71 (53.4)
CCRT	62 (46.6)

*CCRT, concurrent chemoradiotherapy; FIGO, International Federation of Gynecology and Obstetrics; IQR, interquartile range*.

*Data are median (IQR) or n (%)*.

### GI Toxicity and Nutritional Status During Pelvic Radiotherapy

All patients completed the planned pelvic radiotherapy with a median duration of radiotherapy of 39 days (IQR: 37–41). Overall, 28 (21.1%) patients reported PRO-CTCAE score ≥3 for abdominal pain or diarrhea. In all, 14 (10.5%) patients reported severe or very severe abdominal pain, 16 (12.0%) reported that their abdominal pain interfered with their activities quite a bit or very much, and 27 (20.3%) patients reported frequent or almost constant diarrhea.

The nutritional status deteriorated during pelvic radiotherapy with an increase in the PG-SGA score from the start to the end of radiotherapy (1.4 to 3.3, *p* < 0.001). The number of malnourished patients was 13 (9.8%) at the start of radiotherapy and increased to 42 (31.6%) at the end of radiotherapy. Patients with PRO-CTCAE scores ≥3 had significantly higher PG-SGA scores at the end of radiotherapy than those reporting PRO-CTCAE ≤2 (7.6 vs. 2.1%, *p* < 0.001). At the end of radiotherapy, the proportion of malnourished patients was higher in the PRO-CTCAE score ≥3 group than in the PRO-CTCAE score ≤2 group (85.7 vs. 17.1%, *p* < 0.001).

### Skeletal Muscle Changes After Surgery and Adjuvant Radiotherapy

The median duration from pre-treatment CT to simulation CT for radiotherapy and post-treatment CT was 23 days (IQR: 21–25) and 137 days (IQR: 126–144), respectively. The cut-off value for sarcopenia was set at SMI <38.5 cm^2^/m^2^, which corresponds to the lowest tertile. Changes in the BMI and SMI were seen across the three time points (*p* = 0.004 and *p* = 0.02, respectively). BMI decreased from the baseline level by 1.0% post-surgery (23.94 vs. 23.69 kg/m^2^, a decrease of 0.25 kg/m^2^; 95% CI: −0.33 to −0.18; *p* < 0.001), and returned to the baseline level 3 months post-radiotherapy (23.94 vs. 23.95 kg/m^2^, an increase of 0.01 kg/m^2^; 95% CI: −0.17 to 0.18; *p* = 0.95). SMI decreased from the baseline level by 0.4% post-surgery (38.7 vs. 38.5 cm^2^/m^2^, a decrease of 0.2 cm^2^/m^2^; 95% CI: −0.2 to −0.1; *p* < 0.001) and by 1.1% 3 months post-radiotherapy (38.7 vs. 38.3 cm^2^/m^2^, a reduction of 0.4 cm^2^/m^2^; 95% CI: −0.7 to −0.1; *p* = 0.007). The changes in BMI and SMI were correlated (ρ = 0.59; *p* < 0.001) (Supplementary Figure 1). After surgery and adjuvant pelvic radiotherapy, 23 (17.3%) and 32 (24.1%) patients developed ≥5% loss of weight and muscle, respectively.

The changes in BMI and SMI after treatment were not significantly different between patients with or without concurrent chemotherapy (BMI: 0.06% vs. −0.03%, *p* = 0.91; SMI: −0.9 vs. −1.3%, *p* = 0.70).

### Skeletal Muscle Change Based on Patient-Reported GI Toxicity or Nutritional Status

The changes in BMI and SMI after treatment according to the PRO-CTCAE and PG-SGA scores are summarized in Table 2. The frequency of patients experiencing weight or muscle loss was significantly higher in the PRO-CTCAE score ≥3 group than PRO-CTCAE score ≤2 group. Nutritional status at the beginning of radiotherapy was not associated with a change in BMI or SMI after treatment. In contrast, malnourished status at the end of radiotherapy was associated with weight or muscle loss after treatment.

**Table 2:** Body mass index and skeletal muscle index changes by PRO-CTCAE and PG-SGA.

	**PRO-CTCAE score**			**PG-SGA at the start of radiotherapy^a^**			**PG-SGA at the end of radiotherapy^a^**		
**Variable**	**≤2 (*n* = 105)**	**≥3 (*n* = 28)**	***p*-value**	**0–3 (*n* = 120)**	**≥4 (n=13)**	***p*-value**	**0–3 (*n* = 91)**	**≥4 (*n* = 42)**	***p*-value**
BMI change, *n* (%)									
Gain or loss <5%	93 (88.6)	17 (60.7)	0.001	99 (82.5)	11 (84.6)	1.00	88 (96.7)	22 (52.4)	<0.001
Loss ≥5%	12 (11.4)	11 (39.3)		21 (17.5)	2 (15.4)		3 (3.3)	20 (47.6)	
SMI change, *n* (%)									
Gain or loss <5%	94 (89.5)	7 (25.0)	<0.001	93 (77.5)	8 (61.5)	0.30	89 (97.8)	12 (28.6)	<0.001
Loss ≥5%	11 (10.5)	21 (75.0)		27 (22.5)	5 (38.5)		2 (2.2)	30 (71.4)	

*BMI, body mass index; PRO-CTCAE, Patient-Reported Outcome version of the Common Terminology Criteria for Adverse Events; PG-SGA, Patient-Generated Subjective Global Assessment; SMI, skeletal muscle index*.

a *Malnourished defined as PG-SGA score ≥4*.

**Figure 3:**
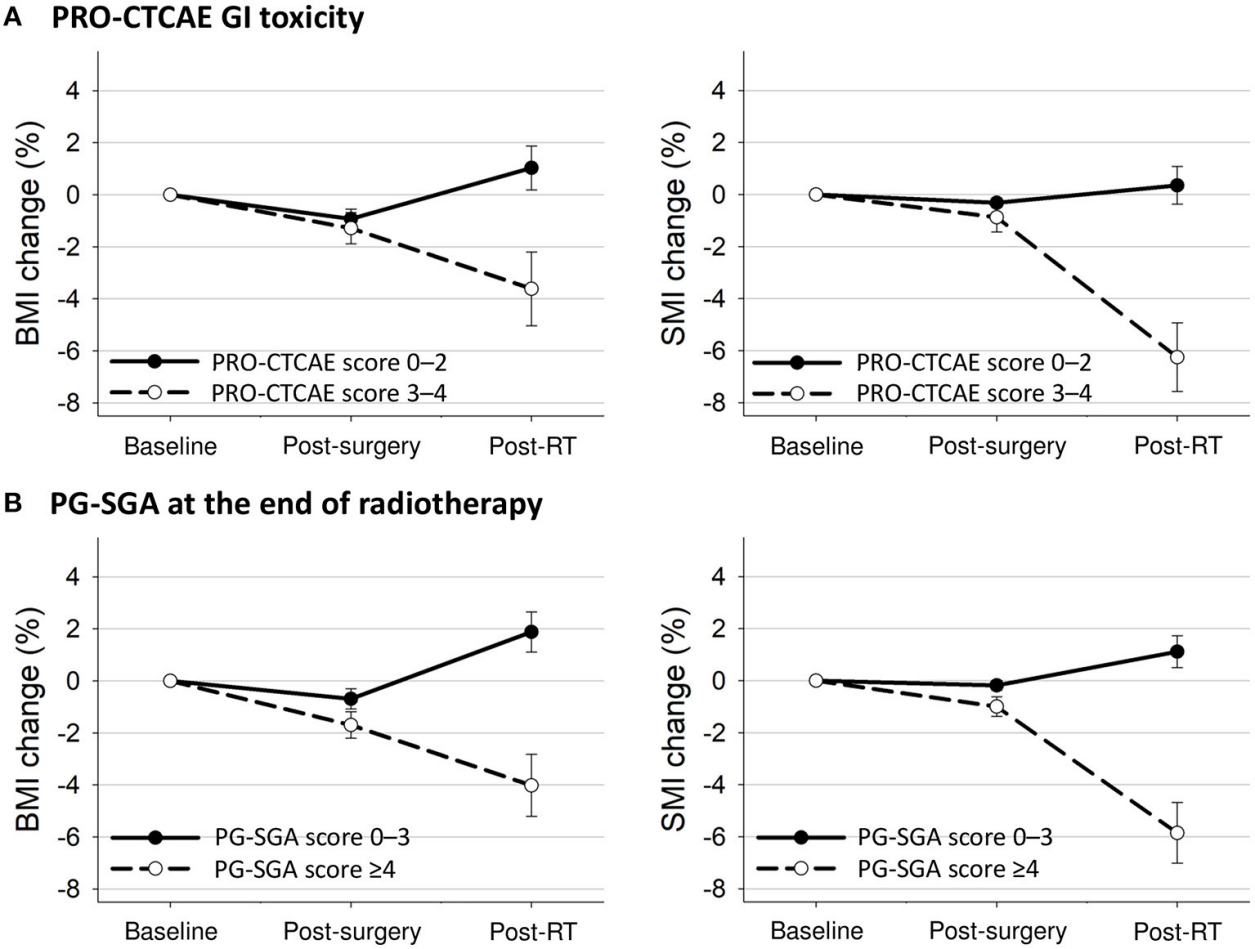
Mean changes with 95% confidence interval bars in BMI and SMI from baseline to 3 months after treatment completion according to **(A)** PRO-CTCAE GI toxicity and **(B)** PG-SGA at the end of radiotherapy. BMI, body mass index; GI, gastrointestinal; PG-SGA, Patient-Generated Subjective Global Assessment; PRO-CTCAE, Patient-Reported Outcome version of the Common Terminology Criteria for Adverse Events; RT, radiotherapy; SMI, skeletal muscle index.

The longitudinal changes in BMI and SMI according to PRO-CTCAE or PG-SGA scores are presented in Figure 3. Patients with PRO-CTCAE score ≥3 showed a greater reduction in SMI after surgery (BMI: −1.4 vs. −0.9%, *p* = 0.26; SMI: −0.9 vs. −0.3%, *p* = 0.04) and in BMI and SMI after radiotherapy (BMI: −3.7 vs. 1.0%, *p* < 0.001; SMI: −6.6 vs. 0.4%, *p* < 0.001) than patients with PRO-CTCAE score ≤2. Patients who were malnourished at the end of radiotherapy had reduced BMI and SMI after surgery (BMI: −1.7 vs. −0.7%, *p* = 0.003; SMI: −1.0 vs. −0.2%, *p* < 0.001) and showed a further decrease in BMI and SMI after radiotherapy (BMI: −4.0 vs. 1.9%, *p* < 0.001; SMI: −5.9 vs. 1.1%, *p* < 0.001) compared to well-nourished patients.

### Prognostic Impact of Skeletal Muscle on Survival

**Figure 4:**
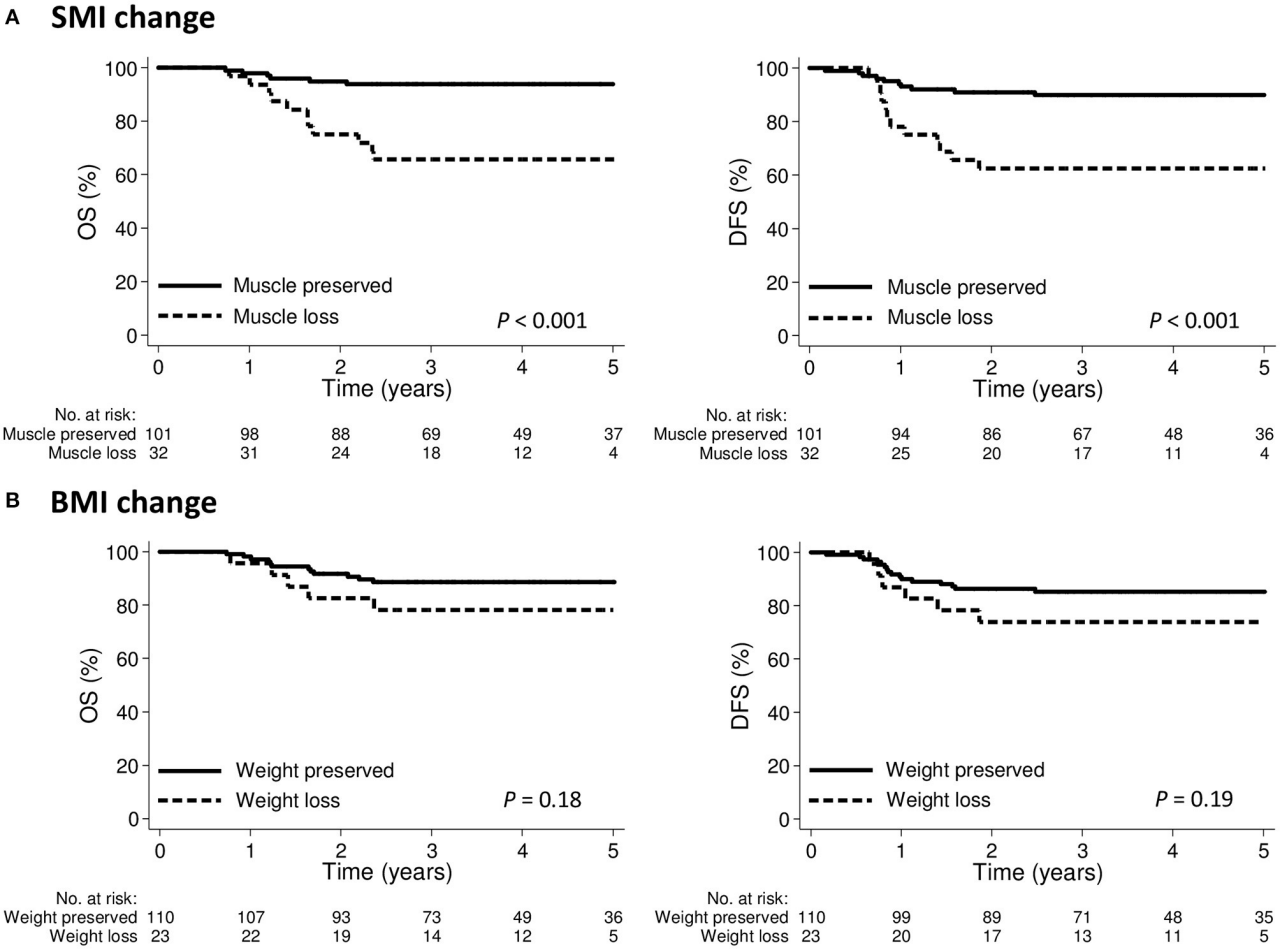
Kaplan-Meier curve demonstrating overall survival and disease-free survival according to **(A)** SMI change or **(B)** BMI change groups. BMI, body mass index; SMI, skeletal muscle index.

The 3-year OS and DFS for the entire cohort were 86.8 and 83.2%, respectively. The 3-year OS was 65.6 and 93.9% in the groups with muscle loss and muscle preserved, respectively (*p* < 0.001); the corresponding 3-year DFS rates were 62.5 and 89.9%, respectively (*p* < 0.001; Figure 4A). The 3-year OS was 78.3 and 88.6% in the weight loss and weight preserved groups, respectively (*p* = 0.18); the corresponding 3-year DFS was 73.9 and 85.2%, respectively (*p* = 0.19; Figure 4B).

On univariate analysis, adenocarcinoma, malnourishment at the end of radiotherapy, pre-treatment sarcopenia, and muscle loss were associated with poor OS and DFS (Table 3). Multivariate analysis showed that adenocarcinoma and muscle loss were independently associated with poor OS and DFS. Malnourishment at the beginning of radiotherapy, pre-treatment BMI, and weight loss after treatment were not associated with OS or DFS. In a subgroup analysis of patients with squamous cell carcinoma (*n* = 104), pre-treatment sarcopenia and muscle loss after treatment were independently associated with poor OS. Muscle loss was independently associated with poor DFS; however, pre-treatment sarcopenia was not associated with DFS (Supplementary Table 1).

**Table 3:** Univariate and multivariate analyses of factors associated with overall survival and disease-free survival.

		**Overall survival**				**Disease-free survival**			
		**Univariate**		Multivariate^*^		**Univariate**		Multivariate^*^	
**Characteristics**		**HR (95% CI)**	***p*-value**	**HR (95% CI)**	***p*-value**	**HR (95% CI)**	***p*-value**	**HR (95% CI)**	***p*-value**
Age	continuous	0.99 (0.94–1.03)	0.55			0.99 (0.95–1.03)	0.64		
FIGO stage	IIA vs. IB	1.09 (0.38–3.08)	0.88			0.78 (0.29–2.12)	0.63		
Histology	AC vs. SCC	4.50 (1.73–11.67)	0.002	3.96 (1.50–10.43)	0.005	2.79(1.19–6.54)	0.02	2.44 (1.04–5.75)	0.04
Pelvc lymph node metastasis	Yes vs. No	0.70 (0.23–2.16)	0.54			1.06 (0.43–2.59)	0.91		
Parametrial involvement	Yes vs. No	1.58 (0.46–5.51)	0.47			1.20 (0.35–4.04)	0.77		
Positive surgical margin	Yes vs. No	2.42 (0.55–10.58)	0.24			1.87 (0.44–8.00)	0.40		
Lymphovascular space invasion	Yes vs. No	0.67 (0.24–1.89)	0.45			0.91 (0.32–2.59)	0.86		
Deep cervical stromal invasion	Yes vs. No	0.91 (0.32–2.59)	0.86			1.02 (0.40–2.62)	0.96		
Adjuvant treatment	CCRT vs. RT	0.81 (0.31–2.13)	0.67			0.96 (0.41–2.22)	0.92		
Malnourished at the start of RT^**^	Yes vs. No	1.98 (0.57–6.88)	0.29			2.10 (0.71–6.22)	0.18		
Malnourished at the end of RT^**^	Yes vs. No	3.15 (1.20–8.28)	0.02			2.25 (0.98–5.19)	0.06		
Pre-treatment BMI	continuous	0.89 (0.78–1.02)	0.10			0.97 (0.87–1.08)	0.53		
Weight loss ≥5% after treatment	Yes vs. No	2.01 (0.71–5.71)	0.19			1.87 (0.73–4.77)	0.19		
Pre-treatment sarcopenia	Yes vs. No	3.04 (1.16–7.99)	0.02	2.67 (0.99–7.17)	0.051	2.13 (0.92–4.92)	0.08		
Muscle loss ≥5% after treatment	Yes vs. No	6.26 (2.31–16.94)	<0.001	4.55 (1.63–12.72)	0.004	4.27 (1.84–9.89)	0.001	3.94 (1.69–9.19)	0.001

*AC, adenocarcinoma; BMI, body mass index; CCRT, concurrent chemoradiotherapy; CI, confidence interval; FIGO, International Federation of Gynecology and Obstetrics; HR, hazard ratio; RT, radiotherapy; SCC, squamous cell carcinoma; SMI, skeletal muscle index*.

* *Multivariable analysis using a backward selection method*.

** *Malnourished defined as PG-SGA score ≥4*.

## Discussion

This study found that skeletal muscle loss after surgery and adjuvant pelvic radiotherapy was associated with poor survival outcomes in patients with early-stage cervical cancer. However, pre-treatment sarcopenia, BMI, and weight loss after treatment were not independently associated with survival outcomes. In addition, skeletal muscle loss was associated with patient-reported GI toxicity and deterioration of nutritional status during pelvic radiotherapy.

The current role of adjuvant pelvic radiotherapy is to decrease the risk of pelvic recurrence in patients with early-stage cervical cancer; however, the outcomes of previous randomized trials indicate that pelvic radiotherapy may not have a benefit of better overall survival (2–4). Pelvic radiotherapy can cause GI toxicity in these patients and deteriorate their nutritional status and quality of life. We found that patients with severe GI toxicities were malnourished at the end of radiotherapy. Severe GI toxicity or malnourishment at the end of radiotherapy was also associated with significant muscle loss after treatment. Notably, patients with muscle loss had significantly poorer OS than those with preserved muscle. Considering the role of adjuvant pelvic radiotherapy mentioned above, we suggest that preservation of muscle mass should be a treatment goal to optimize the OS in these patients.

Skeletal muscle loss is associated with a higher risk of recurrence and overall and cancer-specific mortality in locally advanced cervical cancer (9–11). Although the patients in this study had early-stage cervical cancer, muscle loss was also associated with a higher risk of recurrence and mortality. Moreover, the most common histological type of cervical cancer is squamous cell carcinoma, and its clinical behavior is less aggressive than that of adenocarcinoma (39). In a subgroup analysis of patients with squamous cell carcinoma, muscle loss was associated with a higher risk of recurrence and mortality. This might be because skeletal muscle, as an endocrine organ, regulates the metabolism and inflammation in the entire body. Changes in the metabolic and inflammatory status caused by muscle loss might create a favorable environment for cancer cell growth and disease recurrence (40–42). However, the mechanisms linking muscle loss, recurrence, and cancer-specific mortality need to be investigated in further studies.

Many factors can contribute to muscle loss, including malnutrition, treatment-related toxicity, systemic inflammation, physical inactivity, and aggressiveness of cancer itself (16). In this study, patients with severe GI toxicity or malnourished status at the end of radiotherapy had considerable muscle loss after treatment. Although supportive care such as medication or nutritional counseling was provided to these patients when GI toxicity or malnutrition occurred, there is a need for more effective interventions to preserve skeletal muscle, particularly for patients with PRO-CTCAE score ≥3 or malnourished status at the end of pelvic radiotherapy. Considering that the pathophysiology of muscle loss is multifactorial (43), multimodal interventions (nutrition, exercise, and anabolic medication) might help preserve skeletal muscle. The timing and duration of interventions should also be considered because it can take months to restore rapid muscle loss during cancer treatment (44–46). Moreover, our previous study reported that bowel radiation dose-volume is associated with muscle loss during pelvic radiotherapy (8). It is interesting to classify patients into a lower or higher risk of muscle loss based on patients' conditions and bowel radiation dose-volume and may design targeted multimodal supportive care for patients with a higher risk of muscle loss. Future studies are needed.

Skeletal muscle loss may not be detected by measuring body weight during cancer care. Although the changes in BMI were moderately correlated with changes in SMI in this study, evidence has revealed that changes in the adipose tissue could confound the interpretation of the changes in BMI and mask the detection of muscle loss (18). Moreover, pre-treatment BMI or weight loss after treatment was not associated with survival outcomes in our patients. In previous studies that evaluated patients with locally advanced cervical cancer, the prognostic role of BMI was debatable, while muscle loss was associated with poorer survival outcomes (9–12). These findings suggest the relevance of integrating muscle measurements into clinical practice. In this study, we used CT scans acquired during cancer care to measure skeletal muscle. However, CT scans might not be available for all patients with cervical cancer. This is because MRI might be preferred due to its higher ability to evaluate the local invasion of cervical cancer. The interchangeability of CT-and MRI-derived measurements of the cross-sectional area at superior mesenteric artery level has been reported, suggesting that it might be feasible to evaluate skeletal muscle using MRI (47). Further studies are needed to evaluate the interchangeability of CT and MRI-derived skeletal muscle measurement at the level of L3 in cervical cancer. Our findings also need to be validated in future studies.

This study had some limitations. This is a retrospective investigation with a small number of patients and limited follow-up duration. The sample size of this study was inadequate to draw a firm conclusion (48, 49). Longer follow-up is also needed to provide a more comprehensive view of the effects of skeletal muscle loss on outcomes. Information such as quality of life was not available for analysis owing to the retrospective design of the study. Selection bias and residual and unmeasured confounding factors are also potential limitations of this retrospective study. Despite these limitations, the strength of our study is that patients received very similar treatments, and there were patient-reported outcomes of GI toxicity assessment and nutritional assessment. The treatment outcomes were comparable to those reported in previous studies (4–6).

In summary, our findings showed that skeletal muscle loss after surgery and adjuvant pelvic radiotherapy was independently associated with poor survival outcomes in patients with early-stage cervical cancer. Muscle loss is also associated with GI toxicity and deterioration of nutritional status. While adjuvant pelvic radiotherapy can reduce the risk of pelvic recurrence, it is important to preserve the muscle to optimize survival outcomes for these patients. Future studies are necessary to evaluate whether early multimodal interventions can preserve the muscle in these patients.

## Data Availability Statement

The raw data supporting the conclusions of this article will be made available by the authors, without undue reservation.

## Ethics Statement

The studies involving human participants were reviewed and approved by MacKay Memorial Hospital and Changhua Christian Hospital. Written informed consent for participation was not required for this study in accordance with the national legislation and the institutional requirements.

## Author Contributions

JL, J-BL, and M-HW designed the research. JL and J-BL analyzed data and wrote this manuscript. T-CC contributed in performing the research. Y-TJ contributed in performing the image data analysis. F-JS conducted the statistical analysis. Y-JC revised this manuscript critically for important intellectual content. All authors contributed to the article and approved the submitted version.

## Funding

This work was supported by the Ministry of Science and Technology Taiwan (Grant Number: Contract No. MOST 110-2314-B-195-033).

## Conflict of Interest

The authors declare that the research was conducted in the absence of any commercial or financial relationships that could be construed as a potential conflict of interest.

## Publisher's Note

All claims expressed in this article are solely those of the authors and do not necessarily represent those of their affiliated organizations, or those of the publisher, the editors and the reviewers. Any product that may be evaluated in this article, or claim that may be made by its manufacturer, is not guaranteed or endorsed by the publisher.
