# Supplementary figures and images for: Progressive Skeletal Muscle Loss After Surgery and Adjuvant Radiotherapy Impact Survival Outcomes in Patients With Early Stage Cervical Cancer
Source: Front Nutr. 2022 Jan 20;8:773506. doi: 10.3389/fnut.2021.773506 (PMC8810512; doi:10.3389/fnut.2021.773506)

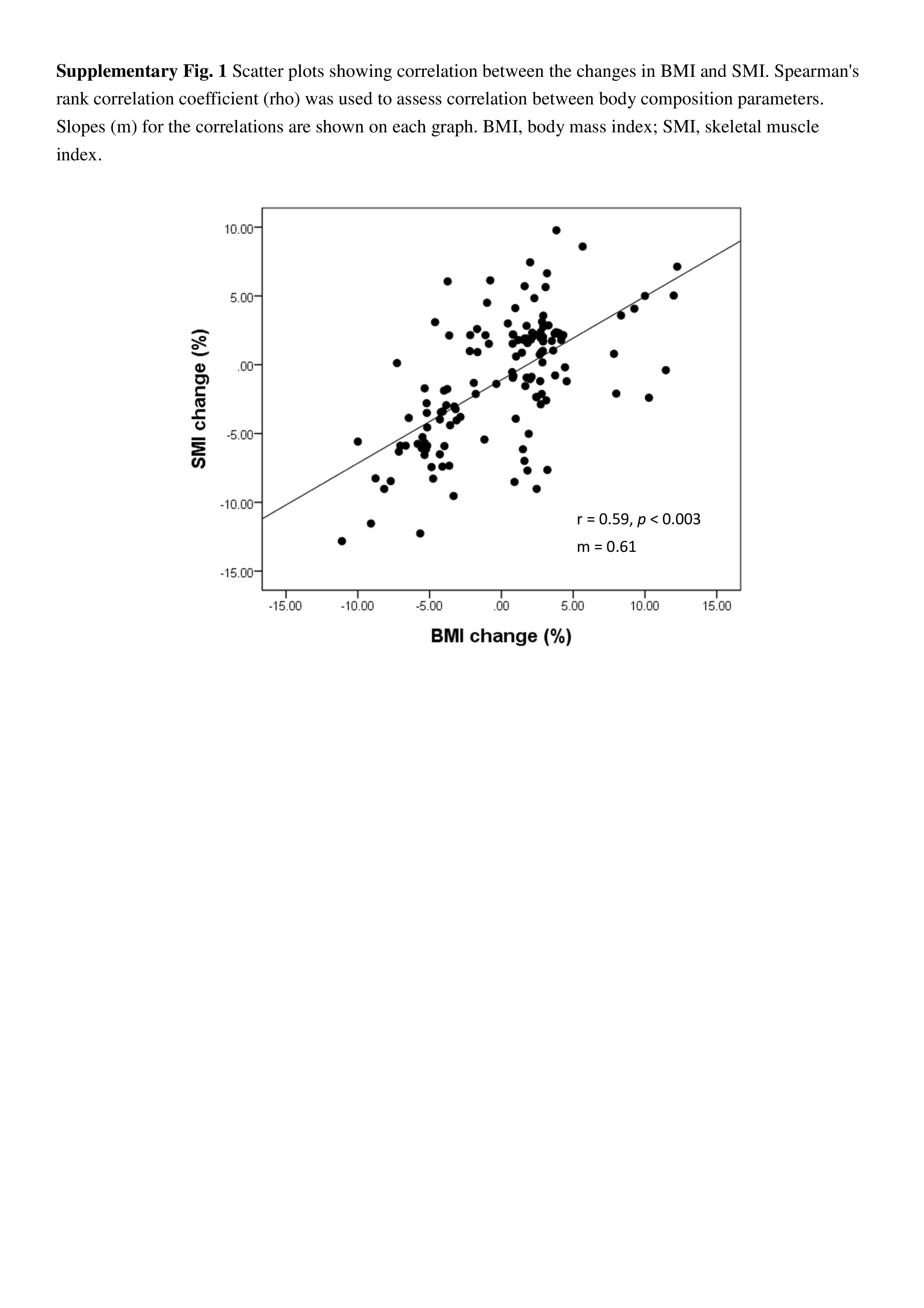

Supplement: Supplementary file 1 [file Image_1.TIF]
