# Supplementary material for: Progressive Skeletal Muscle Loss After Surgery and Adjuvant Radiotherapy Impact Survival Outcomes in Patients With Early Stage Cervical Cancer
Source: Front Nutr. 2022 Jan 20;8:773506. doi: 10.3389/fnut.2021.773506 (PMC8810512; doi:10.3389/fnut.2021.773506)
Supplement: Supplementary file 2 [file Table_1.DOCX]

**Supplementary Table 1** Univariate and multivariate analyses of factors associated with overall survival and disease-free survival is patients with squamous cell carcinoma (n=104).

|  |  | **Overall survival** | | | | |  | **Disease-free survival** | | | | |
| --- | --- | --- | --- | --- | --- | --- | --- | --- | --- | --- | --- | --- |
|  |  | **Univariate** |  |  | **Multivariate*** |  |  | **Univariate** |  |  | **Multivariate*** |  |
| **Characteristics** |  | **HR (95% CI)** | ***p*-value** |  | **HR (95% CI)** | ***p*-value** |  | **HR (95% CI)** | ***p*-value** |  | **HR (95% CI)** | ***p*-value** |
| **Age** | continuous | 0.96 (0.90-1.03) | 0.24 |  |  |  |  | 0.97 (0.92-1.03) | 0.31 |  |  |  |
| **FIGO stage** | IIA vs. IB | 1.54 (0.37-6.45) | 0.55 |  |  |  |  | 0.80 (0.22-2.89) | 0.73 |  |  |  |
| **Pelvc lymph node metastasis** | Yes vs. No | 1.32 (0.32-5.51) | 0.71 |  |  |  |  | 1.92 (0.65-5.72) | 0.24 |  |  |  |
| **Parametrial involvement** | Yes vs. No | 1.03 (0.13-8.39) | 0.98 |  |  |  |  | 0.61 (0.08-4.68) | 0.63 |  |  |  |
| **Positive surgical margin** | Yes vs. No | 2.23 (0.28-18.16) | 0.45 |  |  |  |  | 1.36 (0.18-10.48) | 0.77 |  |  |  |
| **Lymphovascular space invasion** | Yes vs. No | 0.22 (0.05-0.90) | 0.04 |  | 0.24 (0.06-1.03) | 0.054 |  | 0.30 (0.10-0.89) | 0.03 |  | 0.29 (0.10-0.85) | 0.02 |
| **Deep cervical stromal invasion** | Yes vs. No | 0.53 (0.13-2.22) | 0.39 |  |  |  |  | 0.74 (0.23-2.39) | 0.61 |  |  |  |
| **Adjuvant treatment** | CCRT vs. RT | 1.23 (0.31-4.91) | 0.77 |  |  |  |  | 1.45 (0.49-4.30) | 0.51 |  |  |  |
| **Malnourished at the start of RT**** | Yes vs. No | 1.33 (0.16-10.80) | 0.79 |  |  |  |  | 1.73 (0.38-7.80) | 0.48 |  |  |  |
| **Malnourished at the end of RT**** | Yes vs. No | 2.54 (0.64-10.16) | 0.19 |  |  |  |  | 1.60 (0.52-4.88) | 0.41 |  |  |  |
| **Pre-treatment BMI (continuous)** | continuous | 0.83 (0.67-1.02) | 0.08 |  |  |  |  | 0.97 (0.85-1.11) | 0.66 |  |  |  |
| **Weight loss ≥5% after treatment** | Yes vs. No | 3.25 (0.78-13.61) | 0.11 |  |  |  |  | 1.01 (0.13-7.77) | 0.09 |  |  |  |
| **Pre-treatment sarcopenia** | Yes vs. No | 13.46 (1.66-109.44) | 0.02 |  | 9.86 (1.19-81.46) | 0.03 |  | 3.16 (1.03-9.65) | 0.04 |  |  |  |
| **Muscle loss ≥5% after treatment** | Yes vs. No | 6.30 (1.51-26.37) | 0.01 |  | 5.62 (1.33-23.73) | 0.02 |  | 3.36 (1.13-10.02) | 0.03 |  | 3.55 (1.19-10.57) | 0.02 |

Abbreviations: AC, adenocarcinoma; BMI, body mass index; CCRT, concurrent chemoradiotherapy; CI, confidence interval; FIGO, International Federation of Gynecology and Obstetrics; HR, hazard ratio; RT, radiotherapy; SCC, squamous cell carcinoma; SMI, skeletal muscle index.

* Multivariable analysis using a backward selection method.

** Malnourished defined as Patients-Generated Subjective Global Assessment score ≥4.
